# Zinc deficiency associated with anaemia among young children in rural Guatemala

**DOI:** 10.1111/mcn.12885

**Published:** 2019-10-08

**Authors:** Ana M. Palacios, Kristen M. Hurley, Silvia De‐Ponce, Víctor Alfonso, Nicholas Tilton, Kaley B. Lambden, Gregory A. Reinhart, Jeanne H. Freeland‐Graves, Lisa M. Villanueva, Maureen M. Black

**Affiliations:** ^1^ The Mathile Institute for the Advancement of Human Nutrition Dayton Ohio; ^2^ Department of Nutritional Sciences The University of Texas at Austin Austin Texas; ^3^ Center for Human Nutrition, Department of International Health Johns Hopkins Bloomberg School of Public Health Baltimore Maryland; ^4^ Asociación para la Prevención y Estudio del VIH/SIDA Retalhuleu Guatemala; ^5^ Department of Pediatrics University of Maryland School of Medicine Baltimore Maryland; ^6^ Department of Epidemiology and Public Health University of Maryland School of Medicine Baltimore Maryland; ^7^ RTI International, Research Triangle Park North Carolina

**Keywords:** anaemia, hemoglobin, international child health nutrition, micronutrient deficiencies, undernutrition, zinc

## Abstract

One in four children younger than age five in Guatemala experiences anaemia (haemoglobin <11.0 g/dl). This study characterized the factors and micronutrient deficiencies associated with anaemia in a baseline cross‐sectional sample of 182 Guatemalan infants/toddlers and 207 preschoolers, using generalized linear mixed models. Associations between anaemia and maternal, child and household variables, and biomarkers (soluble transferrin receptor, ferritin, zinc, folate, vitamin B12, C‐reactive protein, and α1‐acid glycoprotein) were explored. Rates of anaemia were 56% among infants/toddlers and 12.1% among preschoolers. In children with anaemia, rates of iron deficiency (low ferritin based on inflammation status, and/or high soluble transferrin receptor, ≥1.97 mg/L) and zinc deficiency (serum zinc <65 μg/dl) were 81.1% and 53.7%, respectively. Folate deficiency (either plasma folate <3 ng/ml or erythrocyte folate <100 ng/ml) was 3.3%. Vitamin B12 deficiency (plasma vitamin B12 <148 pmol/L) was 7.5%. For infants and toddlers (<24 months), the odds ratio of anaemia was lower when higher number of adults lived in the household (OR = 0.69; 95% CI [0.53, 0.90]), and higher when children were zinc deficient (OR = 3.40; 95% CI [1.54, 7.47]). For preschoolers (36–60 months), the odds ratio of anaemia was lower for every additional month of age (OR = 0.90; 95% CI [0.81, 1.00]). Findings suggest that micronutrient deficiencies coexist in Guatemalan rural children, and zinc deficiency is associated with anaemia in children <24 months, highlighting the need of continued multidisciplinary interventions with multiple micronutrients. Further research examining how household composition, feeding practices, and accessibility to micronutrient supplements and to animal source foods is needed to incorporate strategies to improve the nutritional status of Guatemalan children.

**Key messages**

- In rural Southwestern Guatemala, anaemia was prevalent in 56% of infants and young children between 6 to 24 months of age and 12% of preschoolers ages 36–60 months. Iron and zinc deficiencies were frequently observed in young children with anaemia, unlike folate and vitamin B12. Zinc deficiency (62.8% of children with anaemia) was associated with anaemia in infants and young children. Historically, local interventions to improve anaemia in Guatemala have focused on iron and vitamin A. Our findings suggest that multiple micronutrient deficiencies exist in young children from rural Southwestern Guatemala.
- In infants and toddlers, anaemia was associated with lower numbers of adults living in the household. This finding suggests that other social factors and caregiver practices may play a significant role in anaemia status. Thus, practices around caregiving should be considered part of the targeted interventions to improve nutritional status and anaemia in the region.

**Abbreviations**
AGP1: α1‐acid glycoprotein
CRP: C‐reactive protein
Hb: haemoglobin
ID: iron deficiency
OR: odds ratio
sTfR: soluble transferrin receptor

## 1 INTRODUCTION

Anaemia is a widespread public health problem that affects more than 2 billion people worldwide (Vos et al., 2016). Anaemia is negatively associated with children's physical and cognitive development, behaviour, and academic performance (Grantham‐McGregor & Ani, 2001; Lozoff, Jimenez, & Smith, 2006; Pollitt, Saco‐Pollitt, Leibel, & Viteri, 1986).

Anaemia has been associated with iron deficiency (ID) and other disadvantages associated with poverty. Recent meta‐analyses and systematic reviews have found mixed findings related to improvement in childhood development after up to 6 months 1α1‐Acid glycoprotein, AGP1; animal source foods, ASF; C‐reactive protein, CRP; haemoglobin, Hb; iron deficiency, ID; months, mo; odds ratio, OR; preschool‐aged children, PSAC; soluble transferrin receptor, sTfR. of iron supplementation/therapy and resolution of anaemia, suggesting that other factors associated with anaemia should be examined (McDonagh, Blazina, Dana, Cantor, & Bougatsos, 2015). Furthermore, children with anaemia or undernutrition have higher morbidity and mortality rates, worse academic performance, and lower earning capacity as adults (Adair et al., 2013; Bhutta, 2013; Rice, Sacco, Hyder, & Black, 2000).

In Guatemala, the latest maternal and child survey in 2014–2015 reported a national anaemia prevalence of 25% among children younger than 5 years of age (MSPAS, 2015), a decline from 47.7% in 2008–2009 (MSPAS, 2008).

Anaemia continues to be an important public health problem, especially among children aged 6 to 11 months, with a prevalence of 64.3%, and those living in rural areas and of indigenous ethnicity seem to be the most vulnerable (MSPAS, 2015).

Although anaemia in Guatemala is poorly characterized, multiple factors have been recognized that are associated with stunting in the region, which is widely prevalent in children <5 years at 46.5% (MSPAS, 2015). Some of the factors associated with stunting in the region include being ages 13–18 months, born at home, Mayan (indigenous) ethnicity, low level of maternal education, short stature, and consumption of iron supplementation (Reurings, Vossenaar, Doak, & Solomons, 2013). The high prevalence of stunting in this country suggests zinc deficiency in children might be widespread (Fischer Walker, & Black, 2007). This is supported by the 2008–2009 national Micronutrient Survey that reported a national prevalence of zinc deficiency of 34.9% (MSPAS, 2012). In the same survey, the prevalence of children with low ferritin (<12 μg/L) and no inflammation (AGP <1 g/L) was of 18.6%.

Currently, little is known to what extent anaemia in young children of rural origin in Southwestern Guatemala is associated with micronutrient deficiencies or other socio‐cultural factors. Yet the rates of anaemia and stunting of children <5 years of the Southwestern region in Guatemala are higher than national average at 31.3% and 51.9%, respectively (MSPAS, 2015).

The purpose of this study was to characterize the factors and micronutrient deficiencies associated with anaemia in a population of infants/toddlers and young children and preschool‐aged rural children from Southwest Guatemala through a cross‐sectional secondary analysis of baseline data from a randomized controlled trial.

## 2 METHODS

### 2.1 Location

The study was conducted in the Municipality of Nuevo San Carlos, in the Department of Retalhuleu, Guatemala. Baseline data were collected between March and May of 2015. According to the National Statistics Institute of Guatemala, Retalhuleu has a population of ~300,000, of which 84.3% is nonindigenous (MSPAS, 2015).

### 2.2 Study design and participants

This cross‐sectional secondary analysis used baseline data collected in a randomized controlled trial. The original trial was designed to examine the effect of a micronutrient supplement and an early learning intervention on the growth, development, and health of infants and toddlers ages 6–24 months and preschoolers from 36 to 48 months with length‐/height‐for‐age *z*‐score <−1, a marker of vulnerability. Children younger than 6 months were not recruited to avoid interfering with exclusive breastfeeding with the nutrition supplement. Inclusion criteria for parents or guardians were age ≥18 years, Spanish speaker, resident in the study community, and not planning to leave the region in the next year. Exclusion criteria for children were severe stunting (length‐ or height‐for‐age *z*‐score <−3 *SD*), severe anaemia (haemoglobin [Hb] <7 g/dl), product of a multiple pregnancy, and genetic or chronic conditions or disability. Children with severe stunting and anaemia were referred to local resources for further evaluation and treatment as indicated. Mothers of participants were informed in advance about the specific date, time, and location of the recruitment and data collection sessions. Data collection was conducted in a community centre with transportation provided.

Based on financial limitations, blood biomarkers were collected from a subsample of 412 participants (189 infants/toddlers and 212 preschoolers), selected through a randomization procedure. Eleven participants did not complete the enrolment survey (four infants/toddlers and seven preschoolers) due to different reasons, nine had severe stunting (five infants/toddlers and four preschoolers), two had severe anaemia (one infant and one preschooler), and one infant had both severe anaemia and severe stunting. The resulting analysis sample included 182 infants/toddlers and 207 preschoolers.

The sample size for the clinical trial was determined by the expected effect size of the intervention (multiple micronutrient fortification powder) on growth and development. The baseline sample size was fixed and no prior studies have shown “true” effect sizes for zinc deficiency and anaemia in a similar population. Thus, post hoc power calculation was not performed using the effect size observed in the analysis, as recommended (Levine & Ensom, 2001).

### 2.3 Data collection

A baseline survey that included family demographics, social, and environmental factors was adapted from previously validated questionnaires and administered to mothers/caregivers at the community centre by local trained field workers (Fernandez‐Rao et al., 2014). Table S1 lists the measurements assessed in the trial at baseline.

### 2.4 Anthropometry

Anthropometry was collected by a standardized team following a detailed protocol (Gibson, 2005).

Weight was measured using the Health‐o‐meter 553KL Digital Portable Pediatric Baby Scale® (Pelstar LLC, Countryside, IL, USA) for infants/toddlers and the digital weight scale Tanita, UM‐028® (Tanita, Chicago, IL, USA), for preschoolers. Recumbent length was measured for infants/toddlers; standing height was measured for preschoolers using Shorr Board® stadiometers (Weigh and Measure LLC, Olney, MD, USA). Anthropometric *z*‐scores were calculated using the 2006 WHO Child Growth Standards.

### 2.5 Maternal, socio‐economic, and home environment variables

To determine household economic resources, a questionnaire listing 28 household items adapted from the 2014 Guatemala National Survey of Living Conditions was implemented (INE, 2014). An assets variable was created by adding all positive answers with higher scores indicating more assets.

Home environment measurements including child care indicators adapted from the Home Observation for Measurement of the Environment Inventories (Bradley & Caldwell, 1977) and The Household Chaos Scale (Matheny, Wachs, Ludwig, & Phillips, 1995) were collected at baseline and used in the analysis.

Maternal employment was defined as mothers working for four or more days per week outside the home.

### 2.6 Dietary diversity

A semiquantitative food frequency questionnaire based on commonly consumed foods during the previous week was adapted and implemented (Rockett et al., 1997). Dietary data were computed into seven food groups (grains, roots, and tubers; legumes and nuts, milk and dairy products; meat, fish, poultry, and organ meats; eggs; vitamin A‐rich fruits and vegetables; and other fruits and vegetables). Each food group was scored as “0” if there was no consumption reported, or as “1” if consumption of at least one food item within that food group was reported. The weekly dietary diversity score for each participant was computed as the sum of the number of positive answers for the food groups consumed in the previous 7 days. Higher scores represented a more diverse diet.

### 2.7 Blood samples and analysis

Fasting venous blood was drawn from the antecubital vein with stainless‐steel needles by a trained phlebotomist in a closed room. Whole blood was collected into trace element‐free, polyethylene tubes. Samples were refrigerated at −8°C immediately after extraction and shipped to the laboratory for further processing the day of collection, following the INCAP laboratory protocol of sample shipping and handling and the procedures recommended by the International Zinc Nutrition Consultative Group (Brown et al., 2004). Ferritin, folate, and vitamin B12 were measured using a chemiluminescence immunoassay method, with the Maglumi 1,000 (SNIBE, Shenzen, China PR). C‐Reactive protein (CRP) and α1‐acid glycoprotein (AGP1) were detected using a nephelometer (BN ProSpec, Dade Behring, Germany). Haemoglobin (Hb) was measured using whole blood on an automated haematology analyser (Hemaray 83 Rayto, Shenzhen, China PR), and soluble transferrin receptor (sTfR) was determined via an in‐house ELISA (30). Serum zinc was assessed by microwave plasma atomic emission spectrometry (MP‐AES 4200, Agilent Technologies, Australia).

Anaemia was defined per World Health Organization standards as Hb <11 g/dl (WHO, 2011).

Subclinical inflammation was defined as CRP >5 mg/L and/or AGP >1 g/L. Inflammation status categories were defined using the above CRP and AGP concentrations: (a) reference (normal CRP and AGP), (b) incubation (elevated CRP and normal AGP), (c) early convalescence (elevated CRP and AGP), and (d) late convalescence (normal CRP and elevated AGP).

Normality of biomarker concentrations was visually inspected using frequency distributions (histograms) and considered normal if the ratios of skewness to its standard error and kurtosis to its standard error were between ±2. Variables that did not follow a normal distribution were log‐transformed.

Corrected values of ferritin, sTfR, and zinc within inflammation groups were calculated by multiplying individual concentrations by their group correction factors. For ferritin, the correction factors were 0.77 (incubation), 0.53 (early convalescence), and 0.75 (late convalescence; Thurnham et al., 2010). For sTfR and zinc, the correction factors were the ratios of the geometric means of the zinc concentrations of the “reference” group to the respective inflammatory group. For sTfR, the natural log‐transformed concentrations were multiplied by 1.40 (incubation), 1.23 (early convalescence), and 0.94 (late convalescence; Rohner et al., 2017). For zinc, the natural log‐transformed concentrations were multiplied by 0.998 (incubation), 1.022 (early convalescence), and 0.990 (late convalescence; Mburu, Thurnham, Mwaniki, Muniu, & Alumasa, 2010). To facilitate interpretation, the results were converted back to the original scale. Cut‐offs for ferritin were determined based on inflammation status as follows: reference, <12 mg/L; incubation and early convalescence, <15 mg/L; and late convalescence, <22 mg/L. The cut‐off used to define elevated sTfR was determined using the geometric mean of children with no subclinical inflammation, no anaemia, and normal ferritin concentrations (1.97 mg/L; J. Allen et al., 1998). ID was defined as either low ferritin or high sTfR. The cut‐off used to define zinc deficiency was of <65 μg/dl. Folates were analysed using both haematologic indicators for defining folate deficiency (either plasma folate <3 ng/ml or erythrocyte folate <100 ng/ml; WHO, 2012). Vitamin B12 deficiency was defined as <148 pmol/L (200 pg/ml; L. H. Allen, 2009).

### 2.8 Statistical analyses

Data were entered into Magpi® (Magpi, Washington, DC, USA), a cloud‐based software, and the database was exported to SPSS® statistical software package Version 24.0 (IBM SPSS Inc., Chicago, IL, USA) for analysis.

Infants/toddlers were analysed separately from preschoolers because anaemia rates, diets, nutritional status, maternal education, and maternal employment status differed between the two samples. To identify maternal, child, and household factors associated with anaemia, a literature review was performed to identify potential variables associated with anaemia. Those variables available from the survey, previously identified in the literature review, were included in a univariate analysis using logistic regressions. At this stage, the same variables were included in both age categories. To increase the precision of the final models, a forward selection method was used to develop a prediction logistic model using the variables identified in the previous step with a *P* value ≤.2 and incorporated into a logistic regression, using a generalized linear mixed model. A final step included micronutrient deficiencies as fixed effects with a random intercept for cluster. Final generalized linear mixed models were adjusted for maternal education, age, and sex, even though age and sex were not significant in the bivariate analysis. Given the low prevalence of folate and vitamin B12 deficiencies, these were not included in the final models; however, the final models were adjusted by the continuous concentrations of folate and vitamin B12.

To further investigate the association of the sociodemographic variables, in particular “number of adults living at home” with anaemia in the final models, a Poisson model was created using the significant variable as dependent and childcare and other sociodemographic indicators as independent variables. The dependent variable was also categorized to identify how many adults would be needed in the household to reduce the odds of anaemia.

Sensitivity analyses included the construction of unadjusted models with individual micronutrient deficiencies as fixed effects.

## 3 RESULTS

### 3.1 Background characteristics

Baseline characteristics of infants/toddlers and preschoolers are listed in Table 1. Children were evenly distributed by sex in both age categories. Mean age was 13 months for infants/toddlers and 45 months for preschoolers. Eighty percent of infants/toddlers and 3.9% of preschoolers were breastfeeding. Introduction of complementary foods occurred around the fifth month of age in both age categories. Weekly dietary diversity scores were lower in infants/toddlers versus preschoolers (5.6 vs. 6.1, *P* < .001). Consumption of meat, fish, poultry, or organ meats at least once during the previous week by age category were infants <12 months = 47.1%; toddlers 12–21 months = 87.5%; preschoolers 36–<48 months = 95.5%; and preschoolers >48 to <60 months = 95.8%. The prevalence of stunting and underweight were both significantly lower among infants/toddlers than preschoolers (25.8% vs. 41.1%, *P* = .002, and 3.3% vs. 13.0%, *P* < .001, respectively). The prevalence of wasting was below 3.5% in both groups.

**Table 1:** Demographic characteristics of infants and toddlers ages 6 to 24 months and preschoolers ages 36 to 60 months from Retalhuleu, Guatemala^a^^,^ b

	Infants/toddlers (*n* = 182)	Preschoolers (*n* = 207)	All (*n* = 389)
	Value	Value	Value
Age (month)	13.2 ± 4.3	45.3 ± 5.6	30.3 ± 16.8
Female sex (%)	55.5	46.4	50.6
Institutional birth (%)	81.3	82.6	82.0
Prenatal checkups	4.3 ± 3.0	4.9 ± 3.2	4.6 ± 3.1
Breastfeeding duration (month)	11.9 ± 4.7	19.9 ± 9.8	16.1 ± 8.8
Age of introduction to complementary foods (month)	5.1 ± 3.0	5.0 ± 3.6	5.0 ± 3.3
Weekly dietary diversity score^c^	5.6 ± 1.5	6.1 ± 0.9	5.9 ± 1.3
Weekly consumption of meat, fish, poultry, and organ meats (%)	70.6	95.6	83.9
Stunted (%)	25.8	41.1	33.9
Underweight (%)	3.3	13	8.5
Wasted (%)	3.3	2.4	2.8
BMI‐for‐age *z*‐score >2 *SD* (%)	1.7	2.4	2.1
Maternal age (year)	26.8 ± 8.1	30.5 ± 7.9	28.8 ± 8.2
Maternal education (secondary or more; %)	30.2	21.4	25.5
Maternal BMI ≥25.0 (%)	43.4	59.4	51.9
Household Food Insecurity Access Scale^d^ (%)	55.2	65.2	60.6
Indigenous ethnicity (%)	12.7	18	15.5
Number of <18 years old living at home	2.9 ± 1.6	3.3 ± 1.6	3.1 ± 1.6
Number of adults living at home	3.1 ± 1.6	3.0 ± 1.4	3.0 ± 1.5
Single, divorced, or widowed (%)	14.8	14.5	14.7
Mothers works ≥4 days/week outside home (%)	6.6	13.5	10.3
Monthly household income, GTQ^e^	1233.8 ± 698.5	1277.2 ± 819.2	1257.0 ± 764.7

a Values are means ± *SD*s unless otherwise indicated.

b Groups significantly different by *t‐* or chi‐square tests.

c FAO/FANTA Household Dietary Diversity Questionnaire and Guidelines.

d FANTA Household Food Insecurity Access Scale (HFIAS) for Measurement of Household Food Access: Indicator Guide (V.3).

e GTQ, Guatemalan Quetzals (1GTQ = 7.65 USD using exchange rate of March 30, 2015).

A larger proportion of mothers in the infants/toddlers group had completed primary school compared with mothers of preschoolers (30.2% vs. 21.4%, *P* = .048).

Overall, more mothers of preschoolers were overweight or obese, compared with mothers of infants/toddlers (43.4% for mothers of infants/toddlers and 59.4% for mothers of preschoolers, *P* = .002). More than 50% of the households reported food insecurity, with a higher rate in mothers of preschoolers (55.2% in infants/toddlers and 65.2% in preschoolers, *P* = .045). Mothers of preschoolers were twice as likely to be employed outside the home compared with mothers of infants/toddlers (6.6% vs. 13.5%, *P* = .025). About 15% of mothers from both infants/toddlers and preschoolers were single, widowed, or divorced. Mean household income of 1,257 GTQ/month (equivalent to 164.3 USD/month; exchange rate of March 30, 2015) was similar in both groups.

### 3.2 Description of anaemia, micronutrient, and inflammation status

The overall prevalence across all age categories of iron and zinc deficiencies were 73.1%; 95% CI [68.0, 78.2] and 36.3%; 95% CI [31.2, 41.4], respectively.

Table 2 provides summary data on the prevalence of micronutrient deficiencies in children with anaemia. More than half of the infants/toddlers had anaemia (56%; 95% CI [48.8, 63.3]) and experienced high rates of iron‐ and zinc‐deficiency anaemia, 82.9%; 95% CI [73.8, 92.0] and 62.8%; 95% CI [52.4, 73.2], respectively.

**Table 2:** Prevalence of micronutrient deficiencies and inflammation status in infants and toddlers ages 6 to 24 months and preschoolers ages 36–60 months with anemia

	Infants/toddlers			Preschoolers			All		
	*n*	%	95% CI (%)	*n*	%	95% CI (%)	*n*	%	95% CI (%)
Anaemia (Hb <11.0 g/dl)	102	56.0	48.8, 63.3	25	12.1	7.6, 16.6	127	32.6	28.0, 37.3
Low ferritin^a^	42	53.8	42.5, 65.2	3	14.3	2.0, 30.6	45	45.5	35.5, 55.4
Elevated sTfR (≥1.97 mg/L)	48	63.2	52.1, 74.3	16	72.7	52.5, 92.9	64	65.3	55.7, 74.9
Iron deficiency (low ferritin and/or elevated sTfR)	58	82.9	73.8, 92.0	15	75.0	54.2, 96.0	73	81.1	72.9, 89.4
Zinc deficiency (<65 μg/dl)	54	62.8	52.4, 73.2	4	18.2	0.7, 35.7	58	53.7	44.2, 63.3
Folate deficiency^b^	3	3.0	−0.4, 6.5	1	4.3	−4.7, 13.4	4	3.3	0.1, 6.5
Vitamin B12 deficiency (<200 pg/ml)	9	9.3	3.4, 15.2	0	0	—	9	7.5	2.7, 12.3
Elevated CRP (>5 mg/L)	12	13.6	6.3, 21.0	5	20.8	3.3, 38.4	17	15.2	8.4, 21.9
Elevated AGP (>1.0 g/L)	37	41.1	30.8, 51.5	8	34.8	13.7, 55.8	45.0	39.8	30.7, 49.0

Abbreviations: AGP, alpha‐1‐acid glycoprotein; CRP, C‐reactive protein; ID, iron deficiency; sTfR, soluble transferrin receptor.

a Low ferritin was determined based on inflammation status as follows: Reference, <12 mg/L; incubation and early convalescence, <15 mg/L; and late convalescence, <22 mg/L.

b Either plasma folate <3 ng/ml or erythrocyte folate <100 ng/ml.

The proportion of ID in infants/toddlers and preschoolers with no anaemia was high, 80.3%; 95% CI [70.0, 90.1] and 65.0%; 95% CI [57.1, 73.0], respectively. Also, infants/toddlers and preschoolers with no anaemia had a prevalence of zinc deficiency of 36.5%; 95% CI [25.2, 47.7] and 24.8%; 95% CI [18.2, 31.5], respectively.

Rates of folate and vitamin B12 deficiency were under 10.0% in infants/toddlers and preschoolers. The inflammation status of infants/toddlers was 60.9% with no active inflammatory process (reference group), 1.2% in the incubation period, 9.9% in early convalescence, and 28% in late convalescence.

Among preschoolers, anaemia prevalence was 12.1%; 95% CI [7.6, 16.6]. Three fourths of preschoolers experienced ID, 18.2%; 95% CI [0.7, 35.7] had zinc deficiency, and 4.3%; 95% CI [−4.7, 13.4] had folate deficiency, and none had vitamin B12 deficiency. The inflammation status of preschoolers was 58.9% with no active inflammatory process (reference group), none in the incubation period, 14.2% in early convalescence, and 26.8% in late convalescence Table 2.

### 3.3 Logistic regression analyses associated with anaemia

**Figure 1:**
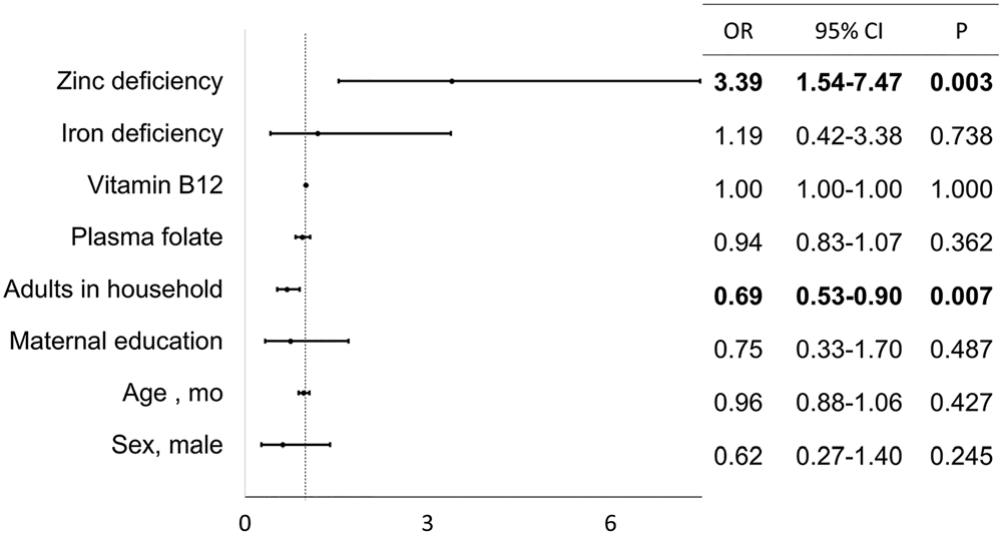
Biomarkers and factors associated with anaemia in infants and toddlers ages 6 to 24 months from Retalhuleu, Guatemala

**Figure 2:**
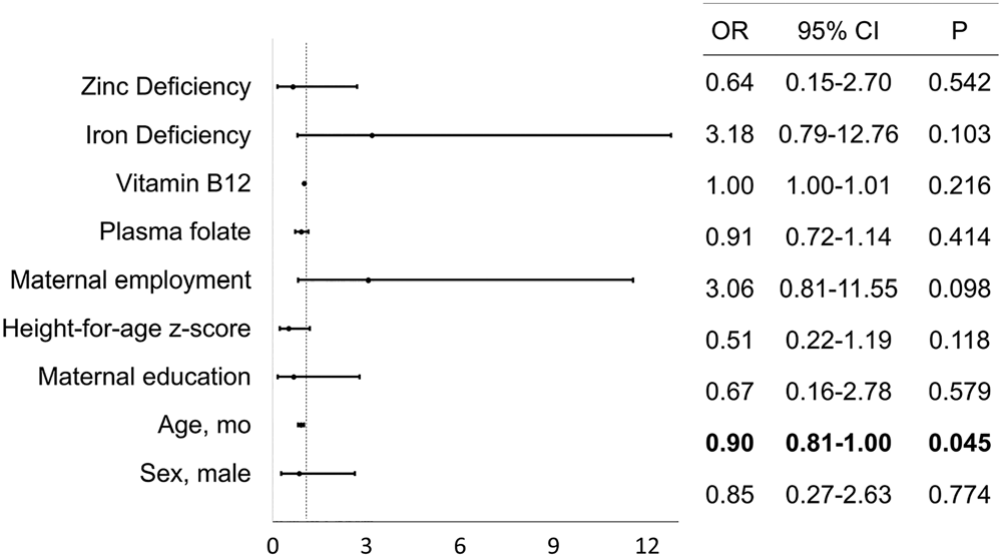
Biomarkers and factors associated with anaemia in preschoolers ages 36 to 60 months from Retalhuleu, Guatemala

Figures 1 and 2 show the results from the multivariate logistic models testing the association between anaemia, blood biomarkers and significant maternal, child, and household factors in infants/toddlers and preschoolers, respectively.

Zinc deficiency was associated with 3.4 times increased odds of anaemia (OR = 3.40; 95% CI [1.54, 7.47]). Child sex, age, maternal education, and ID were not associated with anaemia. Separate models with either iron or zinc deficiency did not show major variability from the reported results. Among infants, the number of adults living at home and zinc deficiency were associated with anaemia. For increasing numbers of adults living at home, the odds of anaemia fell by 31% (OR = 0.69; 95% CI [0.53, 0.90]).

To understand why increasing number of adults living at home was associated with lower odds of anaemia in the infants/toddler group, further Poisson model analysis using “total count of adults living at home” (range = 8) as the dependent variable identified that households with more adults have higher assets scores: (OR = 1.17; 95% CI [0.06, 0.25]). Childcare indicators and other sociodemographic factors were not associated with number of adults living in the household.

Among preschoolers, age was associated with anaemia. For every additional month of age, the odds of anaemia fell by 10% (OR = 0.90; 95% CI [0.81, 0.99]). No other variables were associated with anaemia in preschoolers.

## 4 DISCUSSION

Over half (56%) of infants/toddlers and 12% of preschoolers had anaemia, illustrating a severe public health concern, especially among infants/toddlers. Even though children from this study had a length or height‐for‐age *z*‐score <−1 *SD* at recruitment, findings from this study are close to the age‐related national averages reported in the latest 2014 Maternal and Child Survey in Guatemala (children ages 6–11 months = 64.3%; 12–23 months = 39.6%; 36–47 months = 14.8%; and 48–60 months = 9.1%; MSPAS, 2015).

Micronutrient deficiencies were frequent in both infants/toddlers and preschoolers, with the prevalence of iron and zinc deficiency over 70% and 36%, respectively. ID in infants was comparable with that reported by the 2008–2009 micronutrient survey in Guatemala (80.8% for <12‐month‐old infants with no inflammation). In contrast, the current analysis found a much greater prevalence of ID in preschoolers at 75.0% versus <10.0% (MSPAS, 2012), either due to our selection of children with HAZ <1 or due to an underestimation of ID in the National Micronutrient Survey as they used only ferritin and AGP1. The present study also found low rates of ferritin in preschoolers (14.3%).

The prevalence of zinc deficiency in the current analysis was similar to the rates of the Southwestern region in Guatemala reported by the Micronutrient Survey (36.8%; MSPAS, 2012) and slightly higher than the rates of zinc deficiency from Mexico and Ecuador (27.5% and 28.8%, respectively). It is expected that Guatemala would have higher prevalence of zinc deficiency, as stunting (a functional indicator of zinc deficiency; Fischer Walker, & Black, 2007) is more frequent in Guatemala at ~47%, compared with 14% in Mexico and 29% in Ecuador (Cediel, Olivares, Brito, Cori, & Lopez de Romana, 2015).

In contrast to the lack of association between ID and anaemia, the current study found a strong association between zinc deficiency and anaemia in infants/toddlers. The odds of anaemia were >3 times greater for infants/toddlers with zinc deficiency. To our knowledge, few investigations have examined the relationship between zinc and anaemia. In school‐aged children from New Zealand, zinc deficiency increased the probability of anaemia by five times and with no association between ID and serum ferritin (Houghton, Parnell, Thomson, Green, & Gibson, 2016). A study among preschoolers in Vietnam reported a 45.7% prevalence of zinc deficiency and anaemia, with no association between anaemia and iron status (Van Nhien et al., 2008).

There are several mechanisms potentially linking zinc deficiency and anaemia. Zinc is part of more than 100 enzymes and transcription factors involved in DNA, haem synthesis, and RNA translation, and processes involved in growth, cell division, and erythropoiesis (Labbaye et al., 1995; Nishiyama, Kiwaki, Miyazaki, & Hasuda, 1999). Zinc also is known for stabilizing erythroid cell membranes (O'Dell, 2000) and acts as a catalyst of the enzyme alpha–aminolevulinic acid dehydratase, which modulates erythroid transcriptional gene expression, supports the proliferation of immature erythroblasts, and influences development of erythroid stem cells (Osawa et al., 2002). Also, the lifespan of red blood cells is reduced in the presence of zinc deficiency due to the compromised function of zinc‐dependent antioxidant enzymes (Powell, 2000).

Although no association was found between anaemia and ID, infants/toddlers with or without anaemia and preschoolers showed high rates of ID.

A recent study in India found a weak association between iron intake and anaemia, suggesting that other factors might be playing a more important role in the aetiology of anaemia than previously thought (Swaminathan et al., 2019).

ID occurs when total body iron is reduced, and iron stores are exhausted (Cook, 2005). When haemoglobin concentrations in erythrocytes decrease, their capacity to bind and carry oxygen is jeopardized. This impacts the peripheral tissue oxygenation homeostasis and triggers a series of physiologic responses detailed elsewhere (Weiskopf et al., 1998). Post‐mortem and animal studies suggest that when ID is present, bioavailable iron is prioritized into erythrocytes to favour oxygen delivery over energy production for survival (Petry et al., 1992; Zamora, Guiang, Widness, & Georgieff, 2016). Thus, ID anaemia is thought to be an end‐stage clinical manifestation of ID. These explanations may justify a high prevalence of ID without an association with anaemia as seen in the current study sample.

No biomarkers of nutritional status were associated with anaemia in preschoolers.

Reasons why zinc deficiency was associated with anaemia in infants/toddlers and not in preschoolers may be that infants are particularly vulnerable to micronutrient deficiencies due to their rapid growth, high micronutrient requirements, lower dietary diversity, smaller gastric capacity than older children, and an increased susceptibility to infections leading to gastrointestinal disfunction and environmental enteropathy (Krebs, Miller, & Hambidge, 2014). During the first 4–6 months after birth, iron stores begin to deplete and breastmilk iron and zinc are no longer able to cover the growing infant's needs (Saarinen, Siimes, & Dallman, 1977; Yalcin, Yalcin, & Gucus, 2015). By 7 months, human milk provides only 0.5 mg/day of zinc, or 20% of the estimated average requirement for a 7‐ to 12‐month‐old infant, highlighting the need to incorporate micronutrient‐rich foods as early as 6 months (Krebs, Reidinger, Robertson, & Hambidge, 1994). This analysis identified that less than half of <12‐month‐old infants reported consumption of any meat, fish, poultry, or organ meats at least once during the previous 7 days compared with >95% of preschoolers. Animal source foods are essential for obtaining absorbable forms of iron, zinc, and other micronutrients (Murphy & Allen, 2003) suggesting that infants/toddlers from this region are particularly vulnerable to deficits of some animal‐source essential micronutrients such as iron and zinc. On this matter, there is an opportunity to modify local complementary feeding guidelines as they promote the introduction of beef, fish, and viscera only after 9 months of age (INCAP, FANTA, & Nutri‐Salud/URC, 2016). Other inappropriate complementary feeding practices could be contributing to more prevalent micronutrient deficiencies in infants/toddlers in Guatemala, such as the common habit of providing sweetened water in infants that has been associated with stunting (Doak et al., 2013).

Structural factors and policies also impact the ability of Guatemalan families to access nutrient rich foods. Iannotti and colleagues identified zinc as the micronutrient with the highest probability of inadequacy as income decreased in Guatemala (Iannotti, Robles, Pachón, & Chiarella, 2012). Other cultural and social aspects in the Guatemalan traditional diet can influence complementary food choices in this population. Guatemalan diets are mostly plant based, where the high‐phytate traditional maize (mean phytate content of 840 ± 42 mg/100 g) has been the principal grain of the Mayan people for more than a thousand years and continues to be a main staple (Mazariegos et al., 2006). Phytates form insoluble complexes with divalent minerals (e.g., iron, zinc, and calcium) inhibiting their absorption.

Finally, local efforts to address anaemia through supplementation and fortification have included iron and vitamin A but not zinc.

Among the non‐nutritional findings in this study, we found an inverse association of increasing number of adults in the household with anaemia in infants/toddlers. In low‐ and middle‐income countries, large family sizes have been identified as a factor associated with malnutrition (Keino, Plasqui, Ettyang, & van den Borne, 2014; Pelto et al., 1991), especially when high numbers of children live in the same household (Nkurunziza, Meessen, Van Geertruyden, & Korachais, 2017). In the infant/toddler group, the odds of anaemia fell 31% for each additional adult living in the same household. Yet no association between anaemia and number of children living at home was observed. Further analyses showed that households with more adults had higher asset scores, and mothers of those children also received more education. Consequently, larger families could have a higher socio‐economic status and more resources for children, as well as a stronger social support network, important for the care of infants and toddlers at home. This finding is aligned with a recent cross‐sectional analysis in Uganda that found that larger households had higher incomes, and children living in these households had a lower probability of anaemia (Legason, Atiku, Ssenyonga, Olupot‐Olupot, & Barugahare, 2017).

Uncertainty exists as to how to measure ID in populations. A strength of this study is that it included measurements of inflammation biomarkers (CRP and AGP1) and two iron status indicators: ferritin and sTfR. These measures provide a comprehensive status of ID in this population. The present research is novel in that it is the first in the region to analyze zinc status in the context of anaemia. One limitation is that the sample was selected for infants/toddlers (ages 6–24 months) and preschoolers (ages 36–60 months) with LAZ/HAZ <−1, excluding children with severe stunting or severe anaemia, and therefore is not representative of the population of children younger than age 5 years. Finally, this study did not test for intestinal parasites (amoebas, giardias, and soil‐transmitted helminths) endemic to the region (Hotez, Bottazzi, Franco‐Paredes, Ault, & Periago, 2008) and other secondary and prevalent factors of undernutrition. In the context of this study, households with dirt floors, and lack of access to appropriate sources of water and sanitation, are a reality. In the study location, animals (poultry and other domestic animals) typically inhabit homes where family members eat, cook, and sleep, potentially contributing to the disease burden.

Further research understanding family structures, access to animal source foods, and nutrition supplements could clarify some remaining questions. Developing strategies to increase consumption of iron‐ and zinc‐rich foods in young children is needed. Revising local complementary feeding guidelines, educating health professionals, and implementing culturally relevant education approaches that target caregivers, health care providers, and community leaders are some opportunities to address undernutrition in the region. Also, if food sources of iron and zinc are unavailable or inaccessible, consideration of incorporating iron/zinc supplements during the complementary feeding period would be an inexpensive alternative that could improve the nutritional status of young children in rural Guatemala. Finally, in locations with a high prevalence of stunting and phytate‐rich diets, assessment of zinc should be considered as part of the battery of tests to identify underlying causes of anaemia.

## CONFLICTS OF INTEREST

At the time of the study, AMP, GAR, and LMV worked in the Mathile Institute, which funded the study. The Mathile Institute is a nonprofit organization with no vested interest in the outcomes of this study. The rest of the authors declare no conflict of interest.

## CONTRIBUTIONS

MMB, KMH, and AMP designed the research; SDP, VA, KBL, NT, and LMV conducted the study; AMP, KMH, MMB, and NT analysed the data; all authors contributed to the interpretation of data; AMP, KMH, JFG, and MMB wrote the paper; AMP had primary responsibility for final content. All authors critically reviewed and approved the final manuscript.

## Supporting information

Table S1 Measurements assessed during baseline evaluation
