# Supplementary material for: Zinc deficiency associated with anaemia among young children in rural Guatemala
Source: Matern Child Nutr. 2019 Oct 8;16(1):e12885. doi: 10.1111/mcn.12885 (PMC7038871; doi:10.1111/mcn.12885)
Supplement: Supplementary file 1 — Table S1 Measurements assessed during baseline evaluation [file MCN-16-e12885-s001.docx]

**SUPPLEMENTAL TABLE 1** Measurements assessed during baseline evaluation

| Outcome Variables |  | Measures |
| --- | --- | --- |
| Anthropometry |  | Recumbent length (infants), height (preschoolers), weight, middle upper arm circumference |
| Micronutrient status |  | Blood Hb, ferritin, soluble transferrin receptor, zinc, vitamin B12, serum and erythrocyte folate, CRP, AGP1 and urinary iodine |
| Confounders, moderating or mediating variables | | |
| Maternal |  |  |
| Demographic |  | Education, employment, income |
| Breastfeeding practices |  | Breastfeeding history: initiation, exclusivity and duration |
| Feeding behaviors |  | Infant or preschooler responsive feeding behaviors (Hurley, Black, Papas, Caulfield, & Caufield, 2008) |
| Anthropometric |  | Maternal height and weight |
| Household |  |  |
| Family composition |  | Number of adults and children at home and relation to participant |
| Food security |  | Household food insecurity access scale (Coates, Swindale, & Bilinsky) |
| Dietary |  | Food Frequency Questionnaire (Rockett et al., 1997) |
| Home environment |  | Family care indicators adopted from the Home Observation for Measurement of the Environment Inventories (HOME) (Bradley & Caldwell, 1977)  CHAOS scale (Matheny, Wachs, Ludwig, & Phillips, 1995) |

**REFERENCES:**

Bradley, R. H., & Caldwell, B. M. (1977). Home observation for measurement of the environment: a validation study of screening efficiency. *Am J Ment Defic, 81*, 417-420.

Coates, J., Swindale, A., & Bilinsky, P. Household Food Insecurity Access Scale (HFIAS) for Measurement of Household Food Access: Indicator Guide (V.3). In. Washington, D.C: FHI 360/FANTA.

Hurley, K. M., Black, M. M., Papas, M. A., Caulfield, L. E., & Caufield, L. E. (2008). Maternal symptoms of stress, depression, and anxiety are related to nonresponsive feeding styles in a statewide sample of WIC participants. *J Nutr, 138*(4), 799-805. doi:10.1093/jn/138.4.799

Matheny, A. P., Wachs, T. D., Ludwig, J. L., & Phillips, K. (1995). Bringing order out of chaos: Psychometric characteristics of the confusion, hubbub, and order scale. *Journal of Applied Developmental Psychology, 16*, 429-444. doi:<https://doi.org/10.1016/0193-3973(95)90028-4>

Rockett, H. R., Breitenbach, M., Frazier, A. L., Witschi, J., Wolf, A. M., Field, A. E., & Colditz, G. A. (1997). Validation of a youth/adolescent food frequency questionnaire. *Prev Med, 26*, 808-816. doi:10.1006/pmed.1997.0200
